# Depression and anxiety in patients with rheumatoid arthritis: prevalence rates based on a comparison of the Depression, Anxiety and Stress Scale (DASS) and the hospital, Anxiety and Depression Scale (HADS)

**DOI:** 10.1186/1471-244X-12-6

**Published:** 2012-01-24

**Authors:** Tanya Covic, Steven R Cumming, Julie F Pallant, Nick Manolios, Paul Emery, Philip G Conaghan, Alan Tennant

**Affiliations:** 1 School of Psychology, University of Western Sydney, Australia; 2 Faculty of Health Sciences, University of Sydney, Australia; 3 Rural Health Academic Centre, University of Melbourne, Australia; 4 School of Medicine, University of Sydney, Australia; 5 Leeds Institute of Molecular Medicine, Section of Musculoskeletal Disease, Faculty of Medicine and Health, University of Leeds, UK

## Abstract

**Background:**

While it is recognised that depression is prevalent in Rheumatoid Arthritis (RA), recent studies have also highlighted significant levels of anxiety in RA patients. This study compared two commonly used scales, the Depression Anxiety and Stress Scale (DASS) and the Hospital Anxiety and Depression Scale (HADS), in relation to their measurement range and cut points to consider the relative prevalence of both constructs, and if prevalence rates may be due to scale-specific case definition.

**Methods:**

Patients meeting the criteria for RA were recruited in Leeds, UK and Sydney, Australia and asked to complete a survey that included both scales. The data was analysed using the Rasch measurement model.

**Results:**

A total of 169 RA patients were assessed, with a repeat subsample, resulting in 323 cases for analysis. Both scales met Rasch model expectations. Using the 'possible+probable' cut point from the HADS, 58.3% had neither anxiety nor depression; 13.5% had anxiety only; 6.4% depression only and 21.8% had both 'possible+probable' anxiety and depression. Cut points for depression were comparable across the two scales while a lower cut point for anxiety in the DASS was required to equate prevalence.

**Conclusions:**

This study provides further support for high prevalence of depression and anxiety in RA. It also shows that while these two scales provide a good indication of possible depression and anxiety, the estimates of prevalence so derived could vary, particularly for anxiety. These findings are discussed in terms of comparisons across studies and selection of scales for clinical use.

## Background

Rheumatoid Arthritis (RA) is a multifactorial, chronic, inflammatory disease affecting primarily the joints with prevalence of between 0.5%-1% [1]. Pain, fatigue and disability, which may be considered as stress factors [2], are common challenges that may subsequently lead to psychological distress [3]. Depression commonly co-occurs with RA, in the range of 13% to 20% [4] and above [5] based on clinical assessments. Studies using self report measures of depressive symptoms suggest considerably higher rates (i.e. 40%), although the levels of symptomatology may be subclinical [6]. Longitudinal studies suggest cumulative risk for depression and intermittent recurrence over time [i.e. 40% over 9 years; [7]]. The prevalence rates of depression in RA are well above those reported in the general community or primary care [8] but similar to other chronic conditions [9]. Depression in RA is associated with higher levels of disease activity, pain, fatigue, work disability, health service use but lower treatment compliance [7,10] and increased suicide risk [11,12] and mortality [7,13].

Recent studies have also highlighted significant levels (21% to 70%) of anxiety in RA [5]. While there is a considerable overlap (i.e., 69% shared variance) between anxiety and depression and the levels of anxiety are higher when depression is present [14], the two are considered to be distinctly separate constructs [15]. Some studies report higher prevalence of anxiety than depression in RA [2,16], whereas others do not [5,17]. It is likely that depression and anxiety influence RA via different mechanisms. A recent study found both to have a direct effect on pain, although the direct effect of anxiety was significantly higher than that of depression [18]. While anxiety appeared to increase exposure to stress, only depression influenced pain via stress. According to a tripartite model [15] it is possible that anxiety manifests through physical arousal, increased sensitivity to pain and/or interpretation of sensations as painful. Depression, through the absence of pleasure, may increase vulnerability to pain at times of stress [18] particularly amongst those with repeated episodes of major depression [19]. This explanation is also consistent with a stress process model which suggests that vulnerability factors (such as anxiety and depression) may influence exposure or reaction to stress [20]. Thus, while closely linked, depression, anxiety and stress are proposed to be distinct.

Given the importance of effective screening for depression and anxiety in RA, it is essential to ensure that they are being appropriately measured with available scales. Furthermore, it has been noted that the rates of depression and anxiety vary considerably across studies partly due to differences in assessment scales [5]. Those differences create difficulties in comparing results across studies and, for clinicians, in interpreting with confidence to what degree various scales may identify those who are at risk for depression or anxiety and require referral for further assessment [21].

To examine the degree to which measurements distinguish depression, anxiety and stress, the current study sets out to co-calibrate two commonly used scales. The Depression, Anxiety and Stress Scale [DASS; [22]] and Hospital Anxiety and Depression Scale [HADS; [23]] will be statistically mapped and compared in terms of their construct independence and cut points. The DASS was developed in response to concerns about the potential overlap between depression and anxiety [24], and to independently measure a third factor, stress, which is common to both of those constructs. The *depression *and *anxiety *subscales of the DASS are reported to be consistent with Clark and Watson's [15] constructs of low positive affect and physiological hyper-arousal, although the correspondence of *stress *to their negative affect construct is unclear [25]. Psychometric studies, however, suggest that the DASS measures general psychological distress while still maintaining some distinction between the three separate constructs [26]. A number of factor analytic studies have examined the structure of the DASS, and in general confirmed a three-factor solution [e.g. [27]]. More recently however those traditional methods have been supplemented with modern techniques such as the Rasch measurement model [28]. A recent study used Rasch analysis to assess the short 21-item version of the DASS and found that three items needed to be removed and two subscales, anxiety and stress, appear to measure a single underlying construct [29].

The HADS has been extensively evaluated across various somatic, psychiatric and primary care populations [30] and more recently, musculoskeletal [31], cancer [32], end stage renal disease [33] and RA [21] and found to distinctly measure depression and anxiety with two, seven-item subscales. The HADS was specifically developed for use in primary or secondary health settings to exclude somatic items that may be reflective of the context (i.e. physical condition). It provides a well-established, valid and reliable measure for comparison to the DASS. Both scales have been tested in terms of their screening ability and cut points against commonly accepted diagnostic gold standard such as the Diagnostic and Statistical Manual [33-35]. It is, however, recognised that detection of psychiatric conditions such as depression and anxiety is complicated by the comorbidity of physical conditions even when using diagnostic interviews based on DSM or ICD, unless modified [36,37]. For the purpose of this study HADS will be considered as an interim gold standard as it has been extensively validated across somatic and psychiatric populations in primary care and the general community [30] and is recommended for use in the physically ill [37].

By co-calibrating the items from both scales onto a single metric the three constructs, depression, anxiety and stress, will be mapped to: (a) compare the range of DASS depression and anxiety to that of the HADS scales; and (b) compare the two scales' cut points for depression and anxiety to determine if any variability may be due to scale-specific case definition.

## Methods

### Patients and setting

DASS and HADS item responses were obtained from patients attending the Yorkshire Early Arthritis Register (YEAR) clinic in Leeds, UK (n = 89) and private and hospital clinics in Sydney, Australia (n = 80). All participants met RA diagnosis. Ethical approval was granted by the NHS York Research Ethics Committee, UK (Ref 07/Q1108/25) and the University of Western Sydney Human Ethics Committee, Australia (ref no H5417). Participants' written consent was obtained in accordance with the Declaration of Helsinki.

### Measurements

In addition to demographic data, the following scales were included in the study:

#### Depression Anxiety Stress Scales (DASS)

The Depression Anxiety Stress Scales (DASS) is a 42-item self-report measure of depression, anxiety and stress [22]. It consists of three 14-item subscales with each item scored on a 4-point Likert scale, ranging from 0 (*did not apply to me at all*) to 3 (*applied to me very much, or most of the time*). Total scores are calculated by summing the items on each subscale, giving a score range of 0-42 on each subscale. Scores above 20, 14 and 25 on the depression, anxiety and stress subscales respectively are indicative of severe levels [22]. The DASS shows good convergent and discriminant validity, and high internal consistency and reliability, with Cronbach's alpha reported at 0.94 for Depression, 0.87 for Anxiety and 0.91 for Stress [34].

#### Hospital Anxiety and Depression Scale (HADS)

The Hospital Anxiety and Depression Scale [HADS; [23]] is designed to measure both anxiety and depression in out-patient populations. Each subscale comprises seven items which are rated on a four-point scale and scored from 0-3 with total scores therefore ranging from 0-21 for each subscale. Scores between 0 and 7 represent 'no case'; 8 to 10 indicate 'possible case' and 11 to 21 suggest a 'probable case of anxiety/depression'. These cut points have been validated against clinical interviews with sensitivity and specificity around 0.80 [30]. Recent studies have reported good internal consistency for both anxiety (0.89) and depression (0.86) subscales [38].

### Rasch Analysis

Rasch analysis is a process to test if a set of data intended to be summated to give a total score satisfies the rules for constructing interval scale measurement [39]. The process involves a number of tests to see if the data meet the Rasch model assumptions. These include a probabilistic relationship between items (stochastic ordering); local independence and unidimensionality. The analytical framework also enables checking to see if the scale works in the same way across groups (invariance as determined by Differential Item Functioning-DIF) and examining the reliability and targeting of the scale to the sample. In the context of health outcome measurement the process is described in detail elsewhere [31,40,41].

The process of Rasch analysis is essentially one of testing the above assumptions. Briefly, fit (stochastic ordering) to the Rasch model is achieved when summary and individual item chi-square statistics are non-significant, showing no deviation from model expectation (Bonferroni adjusted); where item and person summary fit statistics show a mean of zero and standard deviation of 1, and where individual item and person residuals are within the range of +/- 2.5.

The assumption of local independence of the item set is verified by the absence of correlations above 0.3 among the residuals of the items tests. Where this assumption is violated and local dependency is found, items can be grouped together in the form of 'testlets', which absorbs the impact of local dependency [42]. The items within each subscale (e.g. depression) can also be grouped together as a testlet and treated as a 'super item' or 'higher order item' [43]. This enables a detailed examination of the relationships between subscales, including the unique component of each scale, the summary correlation between subscales, corrected for attenuation, and the common variance of the total score (adding together all domains assuming unidimensionality) relative to the common and unique variance (i.e. total variance). A high common variance (e.g. 0.9 and above) would indicate that the various subscales were, in general, mapping onto a higher order construct and were correlated sufficiently highly that the total score obtained by summating all subscales together summarizes the profile of the majority of persons [43]. This co-calibration approach also enables a comparison of the operational range of the various subscales, which is one of the aims of the current study.

The test for the assumption of unidimensionality is that described by Smith [44]. Here two estimates are derived from different subsets of items identified by their loading on the first principal component of the residuals. The estimates are compared and when less than 5% are significantly different this supports unidimensionality.

In addition, the scale is expected to show invariance across key groups (e.g. gender or age), as indicated by a non-significant two-way ANOVA of the residuals where group is one factor, and (in this case) the level of psychological distress, a second factor. The latter is determined by simply grouping respondents by their level of distress using the raw score. Reliability indices are also calculated, namely, Cronbach's Alpha and the Person Separation Index (PSI).

The Rasch analysis was conducted using RUMM2030 software [45].

## Results

### Descriptive

In total 169 RA patients were assessed in both countries, and with repeated clinic attendances in the UK, 323 assessments in total were available. The recruitment was designed to give different patterns of disease duration, with the UK data (n = 89) representing early RA (duration < 2 years), and the Australian data (n = 80) more established disease, (mean duration = 13.36, SD = 11.17). However, there were no significant differences in age between the two samples (mean age = 58.3 years, *SD *= 15.1, p >.05), nor a difference in gender (Chi Square 0.059; p = 0.86). For analytical purposes these data were separated into two samples, the initial baseline data of 169 assessments (73% of whom were female), and follow-up data from the UK of 154 repeated assessments (based on two further data collection points).

The mean scores and proportions according to cut points for depression, anxiety and stress (DASS) and depression and anxiety (HADS) are shown in Table 1. While overall mean scores were low across the three subscales on the DASS, 8.3% of the sample recorded scores in the 'severe' or 'extremely severe' range for depression, 7.8% for anxiety and 9.8% for stress. On the HADS, 9.4% of the sample were classified as a 'probable case' of depression (scores 11+) and 18.6% as a 'probable case' of anxiety (scores 11+). Using the 'possible+probable' cut point (scores 8+) from the HADS, 58.3% had neither anxiety nor depression; 13.5% had anxiety only; 6.4% depression only, and 21.8% had both 'possible+probable' anxiety and depression.

**Table 1:** Prevalence of Stress, Anxiety and Depression according to HADS and DASS (n = 169)

Scale	Mean	SD	Possible (H)/Mild & Moderate (D) (%)	Probable(H)/ Severe & Extremely Severe (D) (%)	Poss/Prob(H) Mild, Moderate, Severe/Extremely Severe (D) (%)
Depression (DASS)	6.94	9.22	17.2	8.3	25.5
Depression (HADS)	5.10	4.00	18.9	9.4	28.3
Anxiety (DASS)	5.32	8.01	19.5	7.8	27.3
Anxiety (HADS)	5.97	4.82	16.7	18.6	35.3
Stress (DASS)	9.17	10.49	8.5	9.8	18.3

62.6re/Extreme ut points. ne, with proportions within each actegory. the majoroty that such estimates would be ammenable H = HADS; D = DASS (columns subject to rounding)

### Rasch Analysis

Generally all subscales were consistent with Rasch model expectations, Fit of the baseline data of the individual subscales of the DASS and the HADS, and the various combinations, including the final testlet based version are shown in Tables 2 &3. The DASS-Anxiety subscale showed initial fit to the model (Table 2, Analysis 1), but improved further after some items were rescored (Analysis 2), and local dependency was adjusted by testlets (Analysis 3). The HADS-Anxiety subscale showed good fit at the outset (Analysis 4). All solutions showed strict unidimensionality and local independence. All were free of DIF by age, gender and country. When both the DASS and HADS anxiety items were combined some misfit was observed, and some multidimensionality (Analysis 5). Although creating testlets to accommodate local dependency improved fit (analysis 6), two locally dependent items from the HADS required removal to obtain a strictly unidimensional solution (Analysis 7).

**Table 2:** Fit of the DASS-Anxiety and HADS-Anxiety items to the Rasch model

Analysis	Subscale	Item Fit Residual		Person Fit Residual		Chi-Square Interaction			PSI	Uni-dimensionality	
		Mean	SD	Mean	SD	Value	DF	P		% tests	CI
**Anxiety**											
1	DASS-A	-0.464	1.385	-0.308	0.945	48.21	28	0.010	0.863	6.63	3.3-9.9
2	DASS-A	-0.371	1.352	-0.272	0.970	36.90	28	0.121	0.860	5.42	2.1-8.7
3	DASS-A	-0.219	0.790	-0.267	1.016	16.48	20	0.686	0.839	1.81	-1.5-5.1
4	HADS-A	-0.026	1.509	-0.296	1.088	23.09	14	0.588	0.838	2.41	-0.1-5.7
**Anxiety Combined**											
5	D+H-A	-0.301	1.418	-0.287	1.067	79.648	42	0.0004	0.892	8.38	5.1-11.7
6	D+H-A	-0.152	1.084	-0.296	1.107	42.17	34	0.158	0.880	9.58	6.3-12.9
7	D+H-A	-0.218	1.019	-0.305	1.046	37.11	32	0.245	0.871	4.79	1.5-8.1
**Ideal Fit**		**0.0**	**1.0**	**0.0**	**1.0**			> 0.05*	**> 0.7**	**< 5.0**	**Lower CI below 5**

*Bonferroni adjusted

**Table 3:** Fit of the DASS and HADS items and all subscales (testlets) to the Rasch model

Analysis	Subscale	Item Fit Residual		Person Fit Residual		Chi-Square Interaction			PSI	Uni-dimensionality	
		Mean	SD	Mean	SD	Value	DF	P		% tests	CI
**Depression**											
8	DASS-D	-0.396	1.541	-0.431	1.244	49.69	28	0.007	0.945	6.59	3.3-9.9
9	DASS-D	-0.276	1.567	-0.354	1.132	39.34	22	0.013	0.940	4.79	1.5-8.1
10	HADS-D	-0.263	0.872	-0.382	0.899	25.16	28	0.619	0.856	3.01	0.0-6.3
**Depression Combined**											
11	D+H-D	-0.560	1.422	-0.442	1.133	68.82	42	0.005	0.920	12.05	8.7-15.4
12	D+H-D	-0.423	1.248	-0.373	1.084	57.31	34	0.007	0.912	7.23	3.9-10.5
**Stress**											
13	DASS-S	-0.025	1.383	-0.425	1.575	41.18	28	0.052	0.949	7.19	3.9-10.5
**All Scales combined as testlets**											
14	All	-0.400	0.729	-0.353	0.842	10.73	10	0.378	0.900	2.52	0.0-5.9
15	All	-0.123	0.749	-0.378	0.968	10.12	10	0.430	0.870	2.00	-1.5-5.5
**Ideal Fit**		**0.0**	**1.0**	**0.0**	**1.0**			> 0.05*	**> 0.7**	**< 5.0**	**Lower CI below 5**

*Bonferroni adjusted

The depression subscales showed good fit to the Rasch model, and only the DASS set of items needed adjustment for local dependency (Table 3, Analyses 8 and 9), whereas the HADS-Depression subscale satisfied model expectations from the outset (Analysis 10). All solutions were free of DIF, strictly unidimensional and locally independent. When all items were pooled together, some slight misfit was observed (Analysis 11), but this resolved after creating testlets to accommodate local dependency (Analysis 12). The DASS-Stress subscale also satisfied model expectations at the outset (Analysis 13).

When all subscales were treated as testlets (making five super or higher order items in total), fit to the Rasch model supported unidimensionality of the common latent construct (Analysis 14). The ratio of unique to total non-error variance was high (0.97). Replication was then conducted on the repeated assessment sample (n = 153, UK only) to confirm this interpretation (Analysis 15). The ratio of unique to total variance on this occasion was 0.93, supporting the interpretation that all subscales map on to a common underlying higher-order construct.

**Figure 1:**
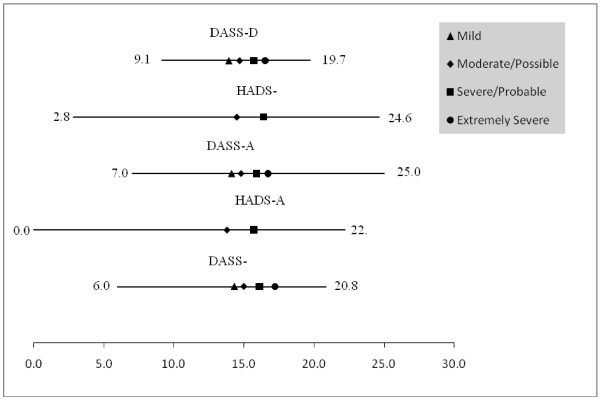
**Operational ranges and cut points of the DASS/HADS subscales**.

The five testlets (i.e. the three DASS subscales and the two HADS subscales, consisting of all items and in their original scoring version) were then mapped, allowing comparison of their operational ranges (Figure 1).

This logit based metric has been rescaled from 0-25 for ease of interpretation, the width of which was determined by a combination of the HADS-Anxiety subscale, which had the lowest level on the common metric (so giving a score of zero), and the DASS-Anxiety subscale which had the highest level on the metric (so giving a score of 25). Both the 'possible' and 'probable' cuts points of the HADS subscales are shown, along with the 'mild' to 'extremely severe' cuts points for the DASS. The HADS-Anxiety subscale has the widest operational range (22.18 on the common metric), and the DASS-Depression subscale the narrowest range (10.61 on the common metric). The cut point for 'extremely severe' on the DASS-Stress subscale is the highest on the construct (so giving the lowest prevalence), followed by the 'extremely severe' cut point for the DASS-Anxiety subscale (Table 4). The HADS-Anxiety 'possible' cut point is the lowest on the common underlying metric, and thus associated with the highest prevalence, giving 35.3% of patients falling above this cut (including the 'probable' patients).

**Table 4:** Hierarchy of various cut points on the common metric*

Scale	Level	Magnitude
DASS-Stress	Extremely Severe	17.16
DASS-Anxiety	Extremely Severe	16.65
DASS-Depression	Extremely Severe	16.52
HADS-Depression	Probable	16.39
DASS-Stress	Severe	16.07
DASS-Anxiety	Severe	15.88
DASS-Depression	Severe	15.68
HADS-Anxiety	Probable	15.68
DASS-Stress	Moderate	15.04
DASS-Anxiety	Moderate	14.78
DASS-Depression	Moderate	14.66
HADS-Depression	Possible	14.53
DASS-Stress	Mild	14.27
DASS-Anxiety	Mild	14.08
DASS-Depression	Mild	13.88
HADS-Anxiety	Possible	13.82

* Scale Ranges: DASS-Stress 5.99-20.83; DASS-Anxiety 7.01-25.0; DASS-Depression 9.13-19.73; HADS-Anxiety 0.0-22.18; HADS-Depression 2.83-24.62.

The HADS-Depression 'probable' cut point and the DASS-Depression 'severe' and 'extremely severe' cut point gave levels of prevalence for depression of a similar magnitude, and are closely located on the underlying metric. However, there is considerable difference in terms of HADS-Anxiety 'probable' when contrasted with the DASS-Anxiety 'severe' and 'extremely severe' cut points, with the prevalence on the HADS-Anxiety more than double that of the prevalence on DASS-Anxiety. This is consistent with the 'probable' cut point on the HADS-Anxiety being lower on the common metric than the DASS-Anxiety 'severe' cut point, thus generating a higher prevalence. However, if the DASS-Anxiety 'mild' cut point is included, then prevalence is similar, with the 'mild' DASS-Anxiety cut point being at a similar location to the HADS-Anxiety 'possible' cut point (Figure 1).

**Figure 2:**
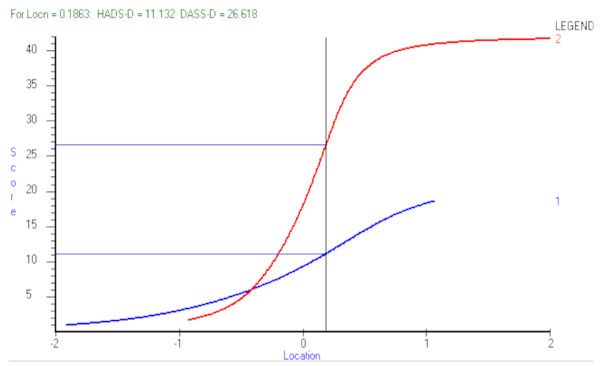
**Co-calibration of HADS 'Probable' Depression Cut Point (1) with DASS**. Read a score of 11 across to the HADS line (1); then read up to the DASS line (2), and then read back to the raw score, which in this example shows 27 (Rounded).

Given the metric, it is possible to provide 'exchange' rates between the respective subscales of the two scales. For example, the 'probable' cut point of the HADS-Depression subscale of 11 points is equivalent to 27 points on the DASS-Depression subscale, which is the highest score within its 'moderate' range (Figure 2).

Likewise the HADS-Anxiety 'probable' cut point of 11 is equivalent to a score of 14 on the DASS-Anxiety subscale, which is the highest score on DASS 'moderate' level. A score of 20 on the DASS-Anxiety subscale (extremely severe) is equivalent to a score of 13 ('probable') on the HADS-Anxiety subscale.

Finally, irrespective of the differential raw scores associated with the various severity-specific cut points on the DASS subscales (e.g. at extremely severe) all severity-specific cut points are shown to map onto a similar point on the underlying metric. That is, the DASS 'extremely severe' cut points for anxiety and depression mark the same location on the underlying metric, as do the 'severe' cut points. It follows from this that the prevalence of the two constructs as defined by the DASS do not vary greatly. The 'extremely severe' cut point for the DASS-Stress subscale is the highest on the metric, as is its 'mild' cut point, compared with the other DASS's subscales, so giving a lower prevalence associated with each cut point.

## Discussion

Depression and anxiety frequently occur in RA. This study compared two commonly used screening scales, HADS and DASS, in relation to their measurement range and cut points to consider if prevalence rates may be due to scale-specific case definition.

The individual subscales of depression and anxiety (HADS and DASS) and stress (DASS only) showed a good fit to Rasch model expectations. After some adjustments to the ordering of thresholds and for local dependency for the DASS Anxiety and Depression subscales, interval scale transformation was achieved for all subscales. When pooled together, DASS Anxiety and HADS Anxiety subscales required some modifications to create a unidimensional scale whereas only minor changes were necessary to merge DASS Depression and HADS Depression. This suggests that the anxiety subscales across the two scales may measure different aspects of this construct. This finding has implications for interpretation of anxiety rates across studies and the selection of anxiety scales. It is important to further investigate the clinical relevance of those scales.

When, however, all subscales were mapped together as testlets, they were found to measure a common underlying trait. This is consistent with the testlet design, when used in this fashion, being equivalent to the bi-factor model, where all sub-domains load onto one common domain, while unique components load on to secondary domains [46]. In this instance it was found that the unique variance was low, and that the majority of variance was shared by the five subscales. This finding may be due to the use of testlets, as in removing local dependency those testlets may also remove some discrete uniqueness within each subscale (which when individually tested all met Rasch analysis expectations). It may also be due to an overlap between the two constructs, depression and anxiety, either as a result of their measures assessing general distress [14] to a degree, or that they may in fact be demonstrating their share common causal factors [47]. Nonetheless, the two constructs are seen as distinctly different, anxiety being motivated by fear while depression by sadness [48]. Finally, the commonality detected may also be due to a high level of comorbidity of depression and anxiety in this study's sample. This comorbidity is particularly significant given its occurrence in the context of a physical comorbidity (RA). It highlights the clinical complexity and associated implications for the treatment of both depression and anxiety in the presence of the physical manifestations of RA. It is also important that further research explores the structure of depression and anxiety constructs in relation to their common underlying construct of psychological distress.

Irrespective of the derivation of the cut points, it was possible through the common metric provided by Rasch analysis, to compare DASS cut points with those of the HADS. Depending upon which cut points were chosen, prevalence estimates could be similar. For example, the HADS 'probable' and DASS 'extremely severe' cut points for depression were found to be closely matched. For anxiety, however, comparable cut points were only found at a lower end. That is, the DASS-Anxiety 'mild' cut point and above matched the HADS-Anxiety 'possible' and above cut point. The DASS-Anxiety 'extremely severe' cut point was higher on the construct than the HADS-Anxiety 'probable' cut point. This may be due to the difference in focus of the anxiety items, with the DASS reflecting feelings of panic and physical symptoms (e.g. dryness of mouth) while the HADS subscale was developed to exclude somatic symptoms and is closely aligned with the generalised anxiety disorder [38]. This is an important finding suggesting that these anxiety scales need further clinical verification to determine which aspects of anxiety they measure, and their concordance with clinical diagnostic assessments.

As such, these findings have important implications for screening and interpretation of research findings across studies. First, this study supports previous findings that elevated levels of both depression and anxiety occur in RA [i.e. [5]]. Second, it permits a direct comparison of the ability of these two measures to distinguish between depression and anxiety, and their derived estimates of prevalence. The operational ranges of measurement between the scales were found to vary and the cut points needed combined calibration in order to determine appropriate comparability.

The DASS cut points were originally determined by distributional difference of their norming sample, later confirmed in clinical populations [34,49,50]. However, in the current study an interesting observation is that the severity-specific DASS cut points for the anxiety and depression subscales all appeared to measure the same point on the underlying metric, irrespective of the specific construct they represent, giving almost identical prevalence for depression and anxiety. In contrast, this was not the case for the HADS. This similarity for DASS cut points requires further investigation.

The study has a number of limitations. Although both scales have previously been evaluated against clinical diagnostic assessment [34,51] it is not possible to determine which one may do so with more specificity/sensitivity in this study. The study was also restricted to RA population, and it would be useful to see if the same results were to be found in other rheumatic diseases given that it is not clear to what degree depression and anxiety presentation and severity may vary across other rheumatic conditions. While the study was not designed to look at responsiveness of these scales, it has shown that their structures are invariant across countries (UK and Australia) and time (examined in UK only). This is an essential requirement for future studies to assess responsiveness of those scales.

In conclusion, the current study highlights the comorbidity of anxiety and depression with RA. It also highlights the challenges of measuring depression and anxiety given the limitations of assessment measures, and the complexity of those conditions. However, this study shows that at least some confusion can be addressed with a co-calibration of depression and anxiety screening scales to enable the comparison of the estimates across scales and studies. That is, the results for the two scales compared in this study suggest that for depression a comparison between the 'probable' category on the HADS and 'extremely severe' on the DASS may be made, but that for anxiety, a comparison of DASS 'mild' and HADS 'possible' is better aligned. This study also highlights the importance of developing a common metric upon which all scales, and their cut points, may be calibrated.

## Conclusions

The authors envisage three key implications from these findings. First, researchers investigating the prevalence of psychological distress amongst people with RA need to be mindful of the lack of concordance of the cut points across scales, and the impact this may have on prevalence estimates and meta-analytic studies. Second, this study reasserts the high rates of depression in RA patients and the need for regular assessment in both early and established RA. This is pertinent given the close association between depression and pain [52] and health outcomes in RA. Third, this study further substantiates converging evidence of the high rates of symptoms of anxiety amongst people with RA. Risk factors for anxiety, unlike for depression, are yet to be systematically examined in relation to RA [53].

In terms of immediate clinical application, screening scales such as HADS and DASS provide a good indication of possible depression and anxiety and may be used for regular screening of psychological distress. It is however, important to understand that those scales may not measure quite the same levels or aspects of that psychological distress, particularly so in relation to anxiety.

## Competing interests

The authors declare that they have no competing interests.

## Authors' contributions

All authors contributed to the conceptualisation of the study. NM, PE and PGC facilitated and coordinated recruitment. AT, TC and JFP analysed the data. TC, AT, SC and JFP drafted the manuscript. All authors read and approved the final manuscript.

## Pre-publication history

The pre-publication history for this paper can be accessed here:

http://www.biomedcentral.com/1471-244X/12/6/prepub
